# Study on the introgression of beef breeds in Canchim cattle using single nucleotide polymorphism markers

**DOI:** 10.1371/journal.pone.0171660

**Published:** 2017-02-09

**Authors:** Marcos Eli Buzanskas, Ricardo Vieira Ventura, Tatiane Cristina Seleguim Chud, Priscila Arrigucci Bernardes, Daniel Jordan de Abreu Santos, Luciana Correia de Almeida Regitano, Maurício Mello de Alencar, Maurício de Alvarenga Mudadu, Ricardo Zanella, Marcos Vinícius Gualberto Barbosa da Silva, Changxi Li, Flavio Schramm Schenkel, Danísio Prado Munari

**Affiliations:** 1 Departamento de Ciências Exatas, Universidade Estadual Paulista (Unesp), Faculdade de Ciências Agrárias e Veterinárias, Jaboticabal, São Paulo, Brazil; 2 Beef Improvement Opportunities (BIO), Guelph, Ontario, Canada; 3 Departamento de Ciências Básicas, Universidade de São Paulo (USP), Faculdade de Zootecnia e Engenharia de Alimentos (FZEA), Pirassununga, São Paulo, Brazil; 4 Departamento de Zootecnia, Universidade Estadual Paulista (Unesp), Faculdade de Ciências Agrárias e Veterinárias, Jaboticabal, São Paulo, Brazil; 5 Embrapa Southeast Livestock, São Carlos, São Paulo, Brazil; 6 Embrapa Agricultural Informatics, Campinas, São Paulo, Brazil; 7 Faculdade de Agronomia e Medicina Veterinária - Universidade de Passo Fundo, Passo Fundo, Rio Grande do Sul, Brazil; 8 Embrapa Dairy Cattle, Juiz de Fora, Minas Gerais, Brazil; 9 Department of Agricultural, Food and Nutritional Science, University of Alberta, Edmonton, Alberta, Canada; 10 Lacombe Research and Development Centre, Agriculture and Agri-Food Canada, 6000 C&E Trail, Lacombe, Alberta, Canada; 11 Department of Animal and Poultry Science, University of Guelph, Centre for Genetic Improvement of Livestock (CGIL), Guelph, Ontario, Canada; Wageningen UR Livestock Research, NETHERLANDS

## Abstract

The aim of this study was to evaluate the level of introgression of breeds in the Canchim (CA: 62.5% Charolais—37.5% Zebu) and MA genetic group (MA: 65.6% Charolais—34.4% Zebu) cattle using genomic information on Charolais (CH), Nelore (NE), and Indubrasil (IB) breeds. The number of animals used was 395 (CA and MA), 763 (NE), 338 (CH), and 37 (IB). The Bovine50SNP BeadChip from Illumina panel was used to estimate the levels of introgression of breeds considering the Maximum likelihood, Bayesian, and Single Regression method. After genotype quality control, 32,308 SNPs were considered in the analysis. Furthermore, three thresholds to prune out SNPs in linkage disequilibrium higher than 0.10, 0.05, and 0.01 were considered, resulting in 15,286, 7,652, and 1,582 SNPs, respectively. For k = 2, the proportion of taurine and indicine varied from the expected proportion based on pedigree for all methods studied. For k = 3, the Regression method was able to differentiate the animals in three main clusters assigned to each purebred breed, showing more reasonable according to its biological viewpoint. Analyzing the data considering k = 2 seems to be more appropriate for Canchim-MA animals due to its biological interpretation. The usage of 32,308 SNPs in the analyses resulted in similar findings between the estimated and expected breed proportions. Using the Regression approach, a contribution of Indubrasil was observed in Canchim-MA when k = 3 was considered. Genetic parameter estimation could account for this breed composition information as a source of variation in order to improve the accuracy of genetic models. Our findings may help assemble appropriate reference populations for genomic prediction for Canchim-MA in order to improve prediction accuracy. Using the information on the level of introgression in each individual could also be useful in breeding or crossing design to improve individual heterosis in crossbred cattle.

## Introduction

Breeding methods using different genetic groups or breeds are very common in animal production, which aims to explore the maximum heterosis on the crossbred individuals as well as for producing composite breeds [1]. In Brazil, the use of composite breeds (also known by the term synthetic breeds) became an important strategy for the beef cattle industry by mixing the intrinsic fitness to the tropical environment by the indicine cattle (*Bos primigenius indicus*) and the higher productive and carcass traits presented by European cattle (*Bos primigenius taurus*) [2]. As an example of a composite breed, which is the focus of our study, the Canchim are resulting from crossbreeding between Charolais and Zebu (mainly Nelore) breeds, resulting in genetic groups with different Charolais-Zebu proportions [2–4].

Canchim cattle has a small inventory of around 30 thousand registered animals, however, this breed has excellent meat yield and quality and performs well when raised on natural pastures of Brazil. The genetic makeup of this composite breed was a 62.5% Charolais and 37.5% Zebu proportion. The genetic group called MA (resulting from mating between Charolais bulls and ½ Canchim + ½ Zebu dams) has an expected genetic makeup of 65.6% Charolais and 34.4% Zebu and is widely used by breeders in order to expand the genetic basis of Canchim [5]. Several studies in this breed have considered different strategies to use the genetic groups for genetic parameter estimation [6], single-marker association [7], genotype imputation [8], and genome-wide association analyses [9]. Other composite breeds, such as Brangus and Girolando, were developed in the South and Southeast of the country, in order to increase meat and milk productivity [1,10].

Beef cattle crossbred populations present animals with different breed compositions. According to Sölkner et al. [11], the introgression of breeds (or admixture) can be measured at the individual animal level through pedigree analysis. However, parental recombination events during meiosis process cannot be estimated using only pedigree information. Therefore, molecular tools, such as single nucleotide polymorphism (SNP) panels, are needed to identify these events and also to provide more accurate information to assist populations under breeding process [12]. According to Frkonja et al. [13], the use of SNPs for admixture studies are more accurate than pedigree analysis.

The complexity of studying composite breeds is due to the occurrence of population stratification, which has a major impact on genomic analyses (i.e. genomic prediction and genome-wide association studies) [14]. The characterization of composite breeds is highly recommended and could aid breeding programs to target specific genotypes of interest. According to Ventura et al. 2016 [15], using clusters of individuals based on their genomic similarities as reference populations could provide better accuracies for genomic predictions in crossbred animals. Thus, the aim of this study was to verify the population structure of Canchim and its genetic groups; and analyze the level of introgression of beef breeds in Canchim using genomic information on Charolais, Nelore, and Indubrasil.

## Materials and methods

This study was approved by the Embrapa Southeast Livestock Ethical Committee for Animal Use (CEUA-CPPSE) under protocol 02/2009 (Canchim, Nelore, and Indubrasil). All Charolais animals were managed according to the guidelines established by the Canadian Council of Animal Care in 1993.

### Breeds and genotype data

All animals except steers from Charolais breed were genotyped with the BovineHD Genotyping BeadChip from Illumina (777,962 SNPs). Charolais steers were genotyped with the Bovine50SNP BeadChip from Illumina (54,609 SNPs). Thus, this study was carried out using the following datasets:

- 285 animals from the Canchim breed (CA) and 114 animals from the MA genetic group (MA) provided by Embrapa Southeast Livestock, located in the municipality of São Carlos, SP, Brazil. Canchim and MA animals were the progeny of 49 bulls and 355 dams, born in seven farms in the states of São Paulo and Goiás between 2003 and 2005.
- 814 animals from the Nelore breed (NE) which are the progeny of 34 bulls, born in the facilities of Embrapa Southeast Livestock and Embrapa Beef Cattle (located in the municipality of Campo Grande, MS, Brazil) between 2007 and 2009.
- 405 steers from the Charolais breed (CH) provided by “Agriculture and Agri-Food Canada Research Centre (AAFC)”, from Canada. Animals were the progeny of 44 bulls, born in the facilities of Onefour Research Ranch, Lethbridge, AB, Canada, and in the Kinsella Research Station, Kinsella, AB, Canada. Animals were born between 2004 and 2009 and a full description was presented by Mao et al. [16].
- 38 animals from the Indubrasil breed (IB) provided by Embrapa Dairy Cattle, located in Juiz de Fora, MG, Brazil. This breed was developed in Brazil from crossings between Gir, Guzerat, and Nelore, all from indicine origin [17].

We highlight that the Charolais, Nelore, and Indubrasil animals herein considered had no direct genetic relationship with Canchim-MA individuals. In Table 1, the accepted crossing schemes to obtain the Canchim breed are presented. In our study, Canchim animals resulting from the crossing scheme I were assigned as CA, while those from the schemes II and III were classified as C1. Canchim animals obtained from the scheme IV were classified as follows: progeny from mating between CA and MA animals were classified as C2 and progeny from mating between MA and MA were classified as C3. This classification resulted in 172 CA, 2 C1, 45 C2, and 66 C3 animals.

**Table 1:** Crossing schemes to obtain animals from the Canchim breed (CA) and other genetic groups through crossings between Charolais (CH) and Zebu (ZB) animals.

**Scheme I**			**Scheme II**		
**Bull**	**x**	**Dam**	**Bull**	**x**	**Dam**
CH (or ZB)	→	ZB (or CH)	CA	→	ZB (or CH)
		↓			↓
ZB	→	½ CH + ½ ZB	CA	→	A
		↓			↓
CH	→	¼ CH + ¾ ZB	CA	→	T1
		↓			↓
5/8 CH + 3/8 ZB	→	5/8 CH + 3/8 ZB	CA	→	V
		↓			↓
		CA			C1
**Scheme III**			**Scheme IV**		
**Bull**	**x**	**Dam**	**Bull**	**X**	**Dam**
CH (or ZB)	→	ZB (or CH)	CA	→	ZB
		↓			↓
CA	→	T2	CH	→	A
		↓			↓
CA	→	V	CA (or MA)	→	MA
		↓			↓
		C1			C2 (or C3)

Adapted from ABCCAN [3] and Alencar [4].

The target animals in our study were Canchim and MA. All other breeds were used for the identification of population stratification and breed composition estimation. Thus, *B*. *p*. *taurus* and *B*. *p*. *indicus* proportions and the contribution from the purebreds were evaluated. By means of pedigree verification, the average expected proportion of *B*. *p*. *taurus* in C1, C2, C3, CA, and MA were 0.57, 0.67, 0.64, 0.62, and 0.65, respectively.

### Data editing and linkage disequilibrium and principal component analyses

Data of animals genotyped with the high-density panel (Canchim, Nelore, and Indubrasil) were extracted to a 50K SNP panel considering the Bovine50SNP map (UMD_3.1 bovine genome assembly). Only autosomal chromosomes and SNPs with known position were used.

The following steps were conducted within PLINK software [18,19] for genotype quality control and merging the files:—cow,—chr 1–29 (autosomal chromosomes),—mind 0.10 (call rate per animal below 0.90 were excluded),—geno 0.05 (call rate for SNP below 0.95 were excluded),—hwe 0.00001 (SNP below this threshold of HW test were excluded),—extract (using Bovine50SNP map), and—merge (merging of genotype data for each breed). The merged genotype data set consisted of 47,782 SNPs and after genotype quality control, 32,308 SNPs (30K) remained for analyses.

Principal component analyses (PCA) were carried out for each breed separately (for Canchim, all genetic groups were included) and considering all breeds together. The function—pca, from PLINK software, followed by the number of principal components desired was used. The PCA were calculated from the genomic relationship matrix and aided in determining clusters of individuals that may or not differ within the breeds. If animals were observed out of the breed main cluster, they were excluded. This exclusion was carried out for CH, NE, and IB breeds. Thus, the final number of animals used for introgression analyses were 395 (CA and MA), 763 (NE), 338 (CH), and 37 (IB).

As the introgression analyses assumes that loci are independent within populations (further explanation in the next section) and that the heterozygosity estimates are sensitive to various ascertainment biases when loci discovered in one breed are used to genotype other breeds [20], we considered pruning the genotypes according to the level of linkage disequilibrium to minimize the effects of these biases [21].

The function—indep-pairwise was used to prune the genotypes, which considered 50 SNPs sliding window and each sliding window moved forward by 10 SNP each time. Three thresholds of linkage disequilibrium (r^2^) higher than 0.10, 0.05, and 0.01 were used to remove SNPs, resulting in three datasets with the number of SNPs of 15,286 (15K), 7,652 (7K), and 1,582 (1K), respectively. The observed and expected heterozygosities for each breed were obtained by the function—hardy of PLINK. The number of SNPs per autosome is presented in Table 2.

**Table 2:** Summary of the number of single nucleotide polymorphisms for the original dataset (50K), after genotype quality control (30K), and after linkage disequilibrium pruning (15K, 7K, and 1K).

Autosome: Length (Megabase pairs)	50K	30K	15K	7K	1K
**1: 158.31**	3,131	2,141	967	486	105
**2: 137.01**	2,552	1,683	763	378	83
**3: 121.39**	2,277	1,562	735	378	78
**4: 120.63**	2,355	1,575	718	375	82
**5: 121.18**	2,050	1,369	628	333	67
**6: 119.42**	2,373	1,664	724	345	72
**7: 112.60**	2,140	1,450	638	315	73
**8: 113.35**	2,179	1,459	669	338	62
**9: 105.67**	1,897	1,355	651	325	63
**10: 104.28**	1,977	1,304	662	315	64
**11: 107.27**	2,057	1,415	657	336	64
**12: 91.12**	1,598	1,035	494	250	56
**13: 84.21**	1,665	1,101	501	249	46
**14: 84.03**	1,686	1,183	505	247	60
**15: 85.23**	1,580	1,080	542	271	63
**16: 81.69**	1,542	1,024	503	252	46
**17: 75.15**	1,442	992	476	223	45
**18: 65.98**	1,248	827	394	195	46
**19: 63.96**	1,274	796	407	213	41
**20: 71.95**	1,407	939	447	223	41
**21: 71.57**	1,312	912	443	222	48
**22: 61.29**	1,193	838	437	208	38
**23: 52.46**	976	682	351	176	36
**24: 62.54**	1,209	778	375	190	37
**25: 42.82**	905	617	315	178	36
**26: 51.64**	1,010	694	352	179	38
**27: 45.40**	895	617	332	158	33
**28: 46.24**	887	588	280	139	24
**29: 51.18**	965	628	320	155	35
**Total**	47,782	32,308	15,286	7,652	1,582

Linkage disequilibrium analyses were carried out for Canchim-MA, Nelore, and Charolais (linkage disequilibrium for Indubrasil presented inflated results due to the low number of individuals and will not be presented). The SNP1101 software, provided by Dr. Mehdi Sargolzaei (University of Guelph), was used for these analyses. The quality control considered the exclusion of minor allele frequency below 0.05 while all the other items described in PLINK were maintained. The linkage disequilibrium (r^2^) was measured as proposed by Hill and Robertson [22] and calculated according to the following equation:

$$r_{ij}^{2}=\frac{p_{ij}-p_{i}\times p_{j}^{2}}{p_{i}\left(1-p_{i}\right)\times p_{j}\left(1-p_{j}\right)}$$ (1)

in which *p*_*ij*_ is the frequency of the two-marker haplotype, and *p*_*i*_ and *p*_*j*_ are the marginal allelic frequencies in the *i*^*th*^ and *j*^*th*^ SNP, respectively [23]. Effective population sizes for Charolais and Nelore at different periods of the population history were estimated by the following equation proposed by Sved [24]:

$$N_{e}=\left(\frac{1}{4_{c}}\right)\left(\frac{1}{r^{2}}-1\right)$$ (2)

in which *Ne* is the effective population size, *c* the marker distance in Morgans (assuming 100,000,000 base pairs per Morgan). The age of *Ne* for any distance was estimated by $\frac{1}{2_{c}}$ [25]. Effective population size was calculated for 1, 5, 10, 20, 50, 100, and 200 generations ago. Due to the recent formation of the Canchim breed and to the small number of genotyped Indubrasil animals, these were not considered for *Ne* estimation.

### Introgression analyses

To detect the introgression of breeds present in CA and MA, the ADMIXTURE [26] software, STRUCTURE [27] software, and the Single Regression method [28] were used. The level of ancestry divergence among populations (*F*_*st*_) was obtained using the ADMIXTURE software in pairwise analyses. The number of populations (k) varied from one to five. The 5-fold cross-validation procedure present in ADMIXTURE was used to define the best k, which should present low cross-validation error when compared to other k values. For the comparison between estimated proportions obtained in these analyses as well as the expected proportions based on pedigree, we calculated the—log of the differences of squared values between each analysis.

#### ADMIXTURE software

In the ADMIXTURE software [26], the likelihood method was applied to estimate the ancestry matrix coefficients. This method considers that the individuals are resulting from random union of gametes and binomial distributions were obtained by:

$$\text{Probability }\left(1/1\text{ for }i\text{ at }SNP_{j}\right)=\;{\left[\Sigma_{k}q_{ik}f_{kj}\right]}^{2}$$ (3)

$$\text{Probability }\left(1/2\text{ for }i\text{ at }SNP_{j}\right)=\;2\left[\Sigma_{k}q_{ik}f_{kj}\right]\;\left[\Sigma_{k}q_{ik}(1-f_{kj})\right]$$ (4)

$$\text{Probability }\left(2/2\text{ for }i\text{ at }SNP_{j}\right)=\;{\left[\Sigma_{k}q_{ik}\left(1-f_{kj}\right)\right]}^{2}$$ (5)

in which allele 1 is the minor allele and the alternative allele 2 is the major allele, *j* is the number of SNP genotypes in *i* unrelated subjects resulting from a composite population with *k* ancestry populations. Population *k* contributes a fraction *q*_*ik*_ of the *i*^*th*^ individual genome. The allele 1 at the j^*th*^ SNP has *f*_*kj*_ frequency in the *k*^*th*^ population.

The method accounts for linkage equilibrium between the markers and the data is recorded as *g*_*ij*_, which represents the observed number of copies of the allele 1 on marker *j* of the individual *i*. Assuming that the individuals are not related, the log likelihood for the full sample id given by:

$$L\left(Q,F\right)=\Sigma_{i}\Sigma_{j}\left\{g_{ij}\;ln\left[\Sigma_{k}q_{ik}f_{kj}\right]+\left(2-g_{ij}\right)\;ln\left[\Sigma_{k}q_{ik}\left(1-f_{kj}\right)\right]\right\}$$ (6)

in which the ancestry matrix coefficients *Q = {q*_*ik*_*}* and the population allele frequencies *F = {f*_*kj*_*}* have *I* x *K* and *K* x *J* dimensions for the total *K(I + J)* parameters. The convergence criteria used for all analyses was 10^−6^, by means of the block relaxation algorithm.

#### STRUCTURE software

The STRUCTURE software [27] considers a Bayesian approach to estimate the ancestry matrix coefficients. The assumptions assumed were: Hardy-Weinberg equilibrium within populations and complete linkage equilibrium between loci within populations. Analyses were performed considering a total of 30,000 iterations and a burn-in period of 10,000 cycles.

In this method, genotypes of the sampled individuals are represented by *X*. The priors considered in the model are: populations of origin of the individuals (unknown) are denoted by *Pr(Z)*, allele frequencies in all populations are denoted as *Pr(P)*, and admixture proportions for each individual are denoted as *Pr(Q)*. The elements of *Q* are *q*_*k*_^*(i)*^, which represents the proportion of individual *i*’s genome that originated from population *k*. The elements of *X* are represented by *x*_*l*_^*(i*,*a)*^, which is the observed allele in the *l*^*th*^ locus for the *i*^*th*^ individual. The elements of *Z* are represented by *z*_*l*_^*(i*,*a)*^, which is equal to the population of origin of allele *x*_*l*_^*(i*,*a)*^.

Thus, the probability model to estimate *P* and *Q* can be represented by:

$$Pr\left(x_{l}^{\left(i,a\right)}=j\;|\;Z,\;P,\;Q\right)=\;p_{zl}^{{\left(i,a\right)}_{lj}}$$ (7)

$$Pr\left(z_{l}^{\left(i,a\right)}=k\;|\;P,\;Q\right)=\;q_{k}^{{\left(i\right)}_{}}$$ (8)

in which *j* is the allele frequency at locus *l* in population *k*. Markov chain Monte Carlo were used for summarizing the information and to obtain the posterior mean for the estimates. The recombination rate was treated as uniform.

#### Single regression method

To predict the breed composition using the regression method (Chiang et al., 2010), genotypes were transformed into copies of allele 1 and divided by 2, resulting in 0, 0.5, or 1. The following single regression model was used:

y=Xβ+e (9)

in which *y* is the transformed genotypes (0, 0.5, or 1) of dimension 32,308 by 1,534, *X* is a matrix of dimension 32,308 by 1 containing the allele frequencies for each breed, *β* is a vector of regression coefficients representing the percentage contribution of each breed to the animal in *y*, and *e* is a random residual vector. Allele frequencies were obtained using the function—freq of PLINK software. Analyses were carried out using the *lm* function present in R software [29].

The regression coefficients were mostly within the range from 0 to 1, however, negative and low values or values higher than 1 were observed. Negative values were coded as 0 and values higher than 1 were coded as 1. The coefficients were adjusted for each individual calculating the product of each regression coefficient with the sum of the regression coefficients. For k = 2 and k = 3, the allele frequencies of Nelore and Charolais; and Nelore, Charolais, and Indubrasil were used, respectively.

## Results

In Table 3, the expected and observed heterozygosity of each breed on the merged genotype data and considering SNP pruning according to the level of linkage disequilibrium is presented. The expected and observed heterozygosities for each SNP density were similar. Results were similar for Nelore and Indubrasil (both indicine breeds) and for Canchim-MA and Charolais (composite and taurine breeds). Canchim and MA, as well as Charolais, presented high heterozygosity for all SNP panels when compared to Nelore and Indubrasil. The high Charolais contribution to Canchim could contribute to the genetic diversity observed in Canchim-MA.

**Table 3:** Mean, standard deviation (SD), and median of the expected and observed heterozygosities in the full genotype data (30K) and in the pruned data according to linkage disequilibrium (15K, 7K, and 1K).

Breed	SNP	Expected heterozygosity		Observed heterozygosity	
		Mean (SD)	Median	Mean (SD)	Median
**Canchim and MA genetic group**	30K	0.26 (0.18)	0.28	0.27 (0.19)	0.28
	15K	0.32 (0.17)	0.38	0.32 (0.18)	0.39
	7K	0.33 (0.19)	0.43	0.33 (0.19)	0.43
	1K	0.36 (0.21)	0.48	0.36 (0.21)	0.47
**Charolais**	30K	0.26 (0.17)	0.28	0.26 (0.18)	0.28
	15K	0.30 (0.17)	0.35	0.31 (0.17)	0.35
	7K	0.31 (0.18)	0.38	0.31 (0.18)	0.38
	1K	0.35 (0.20)	0.47	0.35 (0.20)	0.46
**Nelore**	30K	0.18 (0.18)	0.14	0.18 (0.18)	0.14
	15K	0.25 (0.18)	0.28	0.26 (0.18)	0.28
	7K	0.28 (0.19)	0.34	0.29 (0.19)	0.34
	1K	0.35 (0.20)	0.47	0.35 (0.21)	0.46
**Indubrasil**	30K	0.18 (0.18)	0.10	0.18 (0.20)	0.11
	15K	0.24 (0.19)	0.25	0.25 (0.20)	0.27
	7K	0.27 (0.19)	0.32	0.28 (0.21)	0.32
	1K	0.32 (0.20)	0.41	0.33 (0.21)	0.41

The number of SNP considered from linkage disequilibrium analyses were 37,266 (Canchim-MA), 29,460 (Nelore), and 39,667 (Charolais). In Table 4 are presented the mean r^2^ values for Canchim, Nelore, and Charolais animals, respectively. The effective population sizes for Charolais and Nelore animals for generations 1, 5, 10, 20, 50, 100, and 200, based on the results of linkage disequilibrium analyses, are presented in Table 5.

**Table 4:** Average linkage disequilibrium for Canchim, Nelore, and Charolais animals genomic distance (Megabase pairs—Mb).

Distance (Mb)	Canchim	Nelore	Charolais
**0.00 to 0.02**	0.23	0.23	0.30
**0.02 to 0.04**	0.18	0.19	0.21
**0.04 to 0.06**	0.13	0.16	0.15
**0.06 to 0.08**	0.11	0.13	0.12
**0.08 to 0.10**	0.09	0.11	0.10
**0.10 to 0.20**	0.07	0.09	0.08
**0.20 to 0.30**	0.05	0.06	0.05
**0.30 to 0.40**	0.05	0.05	0.04
**0.40 to 0.50**	0.04	0.04	0.04

**Table 5:** Genome-wide estimates of effective population size from 1 to 200 generations in the past based on estimates of linkage disequilibrium.

Breeds	Generations						
	1	5	10	20	50	100	200
**Charolais**	99	218	295	416	727	1,156	1,759
**Nelore**	116	228	271	371	641	986	1,506

**Fig 1:**
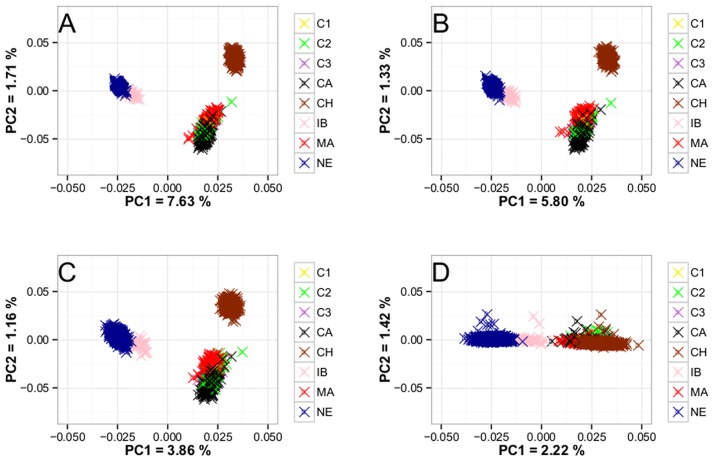
Plot of the first 2 principal components (PC) obtained for the studied breeds. Canchim (C1, C2, C3, and CA), MA genetic group (MA), Charolais (CH), Nelore (NE), and Indubrasil (IB) using 32,308 SNP (A), 15,286 SNP (B), 7,652 SNP (C), and 1,582 SNP (D).

**Fig 2:**
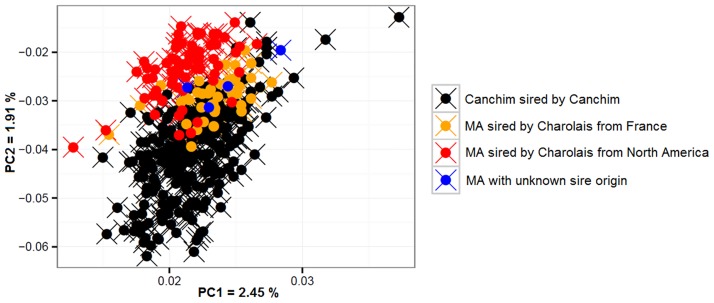
Plot of the first two principal components (PC) obtained for Canchim and MA animals. The plot considered the sire origin.

To characterize the different breeds herein evaluated, principal component analyses were conducted. Fig 1 depicts the PCA plots obtained using the dataset containing 32,308, 15,286, 7,652, and 1,582 SNPs. The percentage of variance (eigenvalues) explained the principal component 1 in the analysis considering 32,308 SNPs was higher than the others. In Fig 2, the PCA from Fig 1 has been highlighted for Canchim and MA animals. The paternal origin of MA animals was from Charolais sires from North America and France.

In the S1 Fig, the cross-validation errors for k from one to five obtained in ADMIXTURE software are presented. According to Alexander et al. [30], a good value of k will exhibit a low cross-validation error compared to other k values. We observed that the highest cross-validation errors (in comparison to the others) were obtained for k = 1. The cross-validation errors for all the other k populations were low. For k = 2, we assumed that the first and second clusters are from *B*. *p*. *indicus* and *B*. *p*. *taurus*, respectively. For k = 3, we assumed that the first, second, and third clusters are from indicine (Nelore), taurine (Charolais), and indicine (Indubrasil) contribution, respectively.

**Fig 3:**
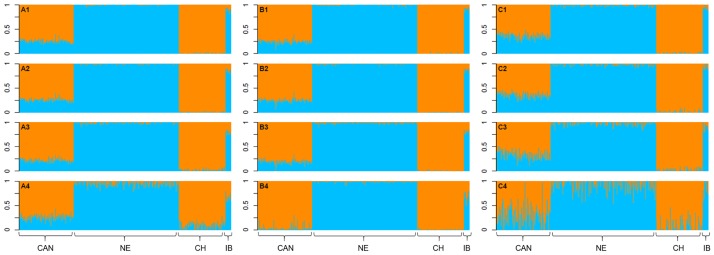
Breed composition per animal considering the number of populations of two (k = 2). Analyses were carried out using ADMIXTURE (A) and STRUCTURE (B) software and Single Regression method (C) and considered 32,308 (1), 15,286 (2), 7,652 (3), and 1,582 (4) SNPs. Orange and blue represents the *Bos primigenius indicus* (cluster 1) and *Bos primigenius taurus* (cluster 2) proportions, respectively. The breeds analyzed were Canchim and MA genetic group together as one breed (CAN), Nelore (NE), Charolais (CH), and Indubrasil (IB).

**Fig 4:**
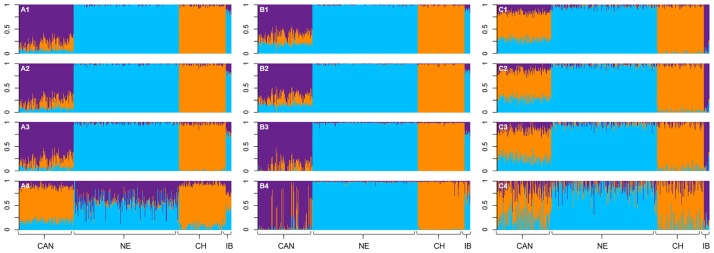
Breed composition per animal considering the number of populations of three (k = 3). Analyses were carried out using ADMIXTURE (A) and STRUCTURE (B) software and Single Regression method (C) and considered 32,308 (1), 15,286 (2), 7,652 (3), and 1,582 (4) SNPs. Blue, orange, and purple represent the Nelore (cluster 1), Charolais (cluster 2), and Indubrasil (cluster 3) proportions, respectively. The breeds analyzed were Canchim and MA genetic group together as one breed (CAN), Nelore (NE), Charolais (CH), and Indubrasil (IB).

The average level of contribution (or ancestry contribution) of *B*. *p*. *taurus* and *B*. *p*. *indicus*, when using k = 2, is presented in S1 Table. The average purebred contributions to Canchim, when using k = 3, are presented in S2 Table. The visual representation of the results obtained using different methods and SNP scenarios are presented in Figs 3 and 4. According to the results observed for ADMIXTURE and STRUCTURE, the estimated proportion of *B*. *p*. *taurus* present in Canchim was approximately 0.75 when k = 2. The average contributions obtained by the Regression method were similar to the pedigree expected proportions for k = 2 and k = 3 in the analysis considering 32,308, 15,286, and 7,652 SNPs (Table 6) in Canchim.

**Table 6:** Comparison between the levels of introgression of breeds present in Canchim-MA animals based on pedigree and prediction methods (ADMIXTURE, STRUCTURE, and Regression). The second cluster of each prediction method was used as a reference for the *Bos primigenius taurus* (Charolais) contribution. The highlighted values in bold were considered highly similar.

**Analyses** ^a^	**Density**	**Pedigree**	**ADMIXTURE (k = 2)** ^b^				**STRUCTURE (k = 2)** ^b^				**Regression (k = 2)** ^b^			
			**30K**	**15K**	**7K**	**1K**	**30K**	**15K**	**7K**	**1K**	**30K**	**15K**	**7K**	**1K**
**Pedigree**	-		1.89	1.88	1.63	1.73	1.86	1.84	1.55	0.96	**2.46**	**2.43**	**2.08**	1.36
**ADMIXTURE (k = 3)** ^b^	30K	0.65		**3.72**	**2.66**	**2.32**	**4.84**	**3.63**	**2.42**	1.29	1.90	1.93	1.93	1.44
	15K	0.67	**3.33**		**2.73**	**2.36**	**3.71**	**4.59**	**2.47**	1.30	1.87	1.92	1.92	1.45
	7K	0.68	**2.77**	**3.06**		**2.29**	**2.72**	**2.82**	**3.56**	1.46	1.63	1.67	1.73	1.41
	1K	1.96	0.51	0.53	0.54		**2.33**	**2.36**	**2.19**	1.34	1.71	1.75	1.78	1.52
**STRUCTURE (k = 3)** ^b^	30K	0.67	**3.65**	**3.42**	**2.88**	0.53		**3.70**	**2.47**	1.31	1.87	1.91	1.91	1.44
	15K	0.64	**3.39**	**3.49**	**2.82**	0.50	**3.08**		**2.54**	1.32	1.84	1.88	1.89	1.45
	7K	0.53	**2.13**	**2.07**	**2.01**	0.41	**2.01**	**2.22**		1.53	1.55	1.58	1.64	1.39
	1K	0.47	1.42	1.38	1.37	0.35	1.39	1.42	1.55		0.96	0.98	1.02	1.06
**Regression (k = 3)** ^b^	30K	**2.03**	0.82	0.84	0.86	1.51	0.85	0.80	0.67	0.59		**3.02**	**2.23**	1.36
	15K	**2.19**	0.77	0.79	0.80	1.62	0.80	0.75	0.63	0.55	**2.94**		**2.34**	1.40
	7K	**2.07**	0.73	0.75	0.77	1.63	0.76	0.72	0.60	0.53	**2.27**	**2.44**		1.42
	1K	1.55	0.62	0.63	0.65	1.51	0.64	0.61	0.51	0.44	1.48	1.54	1.55	

^a^All values were calculated by—log of the differences of squared values between each analysis.

^b^Number of populations of two (k = 2, above diagonal) and three (k = 3, below diagonal).

For k = 2 (S1 Table), the SNP densities of 30k, 15k, 7k, and 1k presented maximum taurine contribution (CL1) equal to 7%, 8%, 11%, and 28% in Nelore when using ADMIXTURE software. Considering the STRUCTURE software, Nelore presented a maximum taurine contribution (CL1) of 8%, 8%, 10%, and 29%. Regression method showed a maximum taurine contribution in Nelore equal to 7%, 10%, 17%, and 59%. Greater maximum values of taurine contribution (CL1) were observed in Indubrasil cattle. Considering k = 3 and the SNP densities of 30k, 15k, 7k, and 1k (S2 Table), the cluster 2 (assigned to the taurine contribution) presented maximum values varying from 2% to 100% for Nelore breed, when using ADMIXTURE and STRUCTURE software. The Regression analysis presented maximum values for cluster 2 of 8%, 12%, 22%, and 47% in the Nelore breed.

Pearson correlations were not adequate to compare the methodologies and SNP densities to estimate the introgression of breeds present in Canchim animals, thus for the comparison between estimated proportions obtained in these analyses as well as the expected proportions based on pedigree were calculated using the—log of the differences of squared values between each analysis. The results of this approach are presented in Table 6, for *B*. *p*. *taurus* contribution (above the diagonal) and for Charolais contribution (below diagonal). We considered an empirical threshold of 2 to explore how the methods behave among each other and with the expected pedigree proportions of *B*. *p*. *taurus* or Charolais.

Similar results between the analyses conducted using 32,308, 15,286, and 7,652 SNP were observed for ADMIXTURE and Structure software. The levels of ancestry divergence (*F*_*st*_) in pairwise analyses are presented in Table 7. The data containing all breeds were analyzed for *F*_*st*_ considering k = 2. For 32,308, 15,286, 7,652, and 1,582 SNPs the *F*_*st*_ estimates were equal to 0.09, 0.08, 0.06, and 0.04, respectively.

**Table 7:** Estimates of the pairwise genetic differentiation statistic (F_st_ statistics) between breeds for SNP^a^ densities of 30K, 15K, 7K, and 1K.

Breeds	Density	Nelore	Charolais	Indubrasil
**Canchim**	30K	0.083	0.049	0.099
	15K	0.063	0.038	0.087
	7K	0.049	0.030	0.088
	1K	0.040	0.046	0.108
**Nelore**	30K	-	0.085	0.048
	15K	-	0.068	0.048
	7K	-	0.051	0.074
	1K	-	0.041	0.173
**Charolais**	30K	-	-	0.109
	15K	-	-	0.106
	7K	-	-	0.103
	1K	-	-	0.116

^a^SNP = single nucleotide polymorphism.

## Discussion

Linkage disequilibrium studies were conducted by Mokry et al. [31], Espigolan et al. [32], and Lu et al. [33] for Canchim-MA, Nelore, and Charolais breeds, respectively. The study by Mokry et al. [31] and Espigolan et al. [32] considered the BovineHD Genotyping BeadChip and presented r^2^ values (above 0.20) up to physical distances of 40 Kb and 30 Kb, respectively. Lu et al. [33] estimated r^2^ for Angus, Charolais, and crossbred individuals (Angus-Charolais) using the Bovine50SNP BeadChip and obtained r^2^ above 0.20 up to 70 Kb, for Angus, and up to 30 Kb, for Charolais and crossbred individuals. In our study, we observed r^2^ values at a longer physical distance only for Charolais (40 Kb), while the other breeds presented r^2^ above 0.20 up to 20 Kb (Table 4). The effective population size in Charolais and Nelore presented a similar number of animals that contributed over the generations, decreasing over time due to breed formation, inbreeding, and artificial breeding techniques.

The observed heterozygosity was obtained to verify the genetic diversity on a global scale and, according to López Herráez et al. [34], SNPs in high linkage disequilibrium could bias the observed heterozygosity and suggested that the removal of these SNPs may control the effect of the bias and generate reliable comparisons between populations. Higher differences for the level of genetic diversity were observed for the 30K and 15K panels when comparing Canchim-MA and Charolais with Nelore and Indubrasil. Porto-Neto et al. [35] observed higher heterozygosity for the composite breeds (0.34) in comparison with the taurine (0.28) and indicine (0.26) breeds. These authors attributed a higher linkage disequilibrium in taurine breeds to a smaller effective population size and a stronger bottleneck during breed formation.

The Fig 1A, 1B and 1C were very similar and, as the density of SNP decreases, the clusters of individuals per breed appear to be sparser. Thus, if very few SNPs were used (Fig 1D) the detection of clusters of individuals could be compromised. It was also noted a higher dispersion in the cluster formed in Canchim and MA groups due to the nature of the composition of the breed. The plasticity of Canchim can be observed by the dispersion of the individuals. In Fig 1, Canchim and MA (black and red dots) diverged into two close clusters. MA animals were traced back to verify the origin of the sire. Charolais sires from North America and France are often used to produce the Canchim (or MA), which could be the reason of the two main clusters observed (Fig 2). Thus, Canchim and its genetic groups presented a clear population stratification, which has to be taken into consideration when analyses, such as genome-wide association and genomic selection, are to be conducted.

In the S1 Fig, we observed that the genetic structure of the studied breeds could not be assigned to only one population. The cross-validation errors were lower for k = 5, however, due to difficulties in the biological explanation for k = 4 and k = 5, we considered discussing the remaining k populations.

The crossing scheme IV (MA, C2, and C3 animals), which produce animals with expected higher *B*. *p*. *taurus* proportion based on pedigree information also presented the highest *B*. *p*. *taurus* proportion based on introgression analyses up to 15,286 SNPs (S1 Table). Furthermore, for k = 2 we can observe similar behavior for all methods up to 7,652 SNPs. The maximum likelihood (ADMIXTURE) method presented a better representation of the expected breed proportions when using lower threshold for linkage disequilibrium (Fig 3–A4), while Bayesian method (STRUCTURE) and Regression method seemed to, respectively, underestimate (Fig 3–B4) and overestimate (Fig 3–C4) the ancestry contribution according to the expected proportion. Additionally, as the density of SNPs decreased more noise has been observed in the purebred individuals. In practical terms, Pritchard et al. [27] pointed out that in admixture analyses it is important to consider how many populations are most appropriate for interpreting the data and then examine the clustering of individuals for the inferred number of populations.

As observed by O’Brien et al. [36], the level of taurine introgression in Nelore is low. The same result was found in our study; however, we applied the PCA filter to remove animals that were not in the main cluster. Thus, these animals, which were not considered in the admixture analyses, might present some higher level of taurine introgression. The same rationale goes for Indubrasil animals, although this breed had a contribution of other indicine breeds, it might have crossed with taurine cattle in the early development of the breed. Furthermore, a less controlled mating scheme at the early stages of Indubrasil development, mainly on the founder breeds (Gir, Guzerat, and Nelore), could contribute to the higher taurine proportion herein observed.

For k = 3 (S2 Table and Fig 4), the ADMIXTURE and STRUCTURE software weren’t able to cluster Indubrasil due to a probable similarity with Nelore. As the Indubrasil present contributions of genes from other indicine breeds, it is understandable that confounding effects with Nelore may occur. In Fig 1, PCA assigned Nelore and Indubrasil breeds into a close cluster, which could be difficult to discriminate both breeds in ADMIXTURE and STRUCTURE software. In general, the PCA and admixture analyses placed the animals into similar clusters of breeds and Canchim and its genetic groups were placed closer to Charolais individuals. Despite the noise observed in the purebreds, the Regression method presented plausible results from a biological point of view, because it separated the data into three main clusters assigned to each purebred animals as well as the contribution of each breed in Canchim and MA animals. It is not clear how Indubrasil may be contributing to Canchim genetic composition since the breed is not presently used to produce the Canchim. However, Indubrasil was largely used for the initial development of Canchim.

As observed by Ardlie et al. [37], recombination events are greatly influenced by the amount of information on the history of population size, gene flow between other breeds, and selection. Furthermore, the recombination is not uniform across a chromosomal segment, rather recombination events tend to occur in recombination hotspots [38] Thus, recombination events could be assigned to the variation observed in the estimated proportions based on introgression analyses.

Low *F*_*st*_ values are related to low differentiation between breeds, which can be observed between Canchim and Charolais (0.049, 0.038, and 0.030) up to linkage disequilibrium pruning of 0.05 (Table 7). The *F*_*st*_ estimates for Nelore and Indubrasil were also low (0.048) up to linkage disequilibrium pruning of 0.10. Higher differentiation was observed between Canchim and Indubrasil (0.099, 0.087, 0.088, and 0.108) than those observed with Nelore because Indubrasil had a former contribution to Canchim. Thus, the level of differentiation between *B*. *p*. *taurus* and *B*. *p*. *indicus* was higher when considering the analysis of 32,308 SNPs.

This is the first work using genomic information to characterize the levels on introgression of beef breeds in Canchim cattle. The methods evaluated could also be suitable for inferring and discriminating animals without pedigree information by using genotype information to assign animals to correct the clusters based on the level of breed introgression. The results herein obtained could provide great information to future studies, such as genomic selection and design of breeding schemes in order to increase or maintain variability. As observed by Ventura et al. [15], genotype clustering methods used together with genomic prediction strategies could be used to build a better reference population in an efficient manner, resulting in more accurate genomic selection predictions for crossbreed data. Canchim was developed to join the adaptability to tropical conditions from the indicine with the precocity and meat quality from the taurine cattle. Therefore, from a practical point of view, the study of introgression in this breed can assist to verify a desirable combination of breeds to obtain adapted animals with higher meat quality.

## Conclusions

Principal component analyses helped to determine clusters of breeds as well as the proximity of Canchim and MA to Charolais. In MA genetic group, individuals were assigned to different clusters due to sire country of origin. Assign our analyses into two clusters (k = 2) seems to be more appropriate for analyzing Canchim-MA animals due to its biological interpretation (regarding the genetic makeup of the breed). Moreover, the usage of 30K density in the analyses presented similar results between the estimated and expected genetic proportions of introgressed breeds in Canchim-MA. In the Regression approach, a remote contribution of Indubrasil was observed in Canchim-MA when three clusters were considered (k = 3).

Genetic parameter estimation in Canchim-MA could account for the level of each introgressed breed as a source of variation in order to improve the accuracy of genetic models. The results of this study may assist to assemble appropriate genomic prediction training population for Canchim and MA animals in order to improve prediction accuracy in genomic selection. Using the information on the level of introgression in each individual could help in breeding or crossing design to improve individual heterosis in Canchim cattle and other Brazilian composite breeds.

## Supporting information

S1 Table Average breed composition for Canchim (C1, C2, C3, and CA), MA genetic group (MA), Charolais (CH), Nelore (NE), and Indubrasil (IB) considering the number of populations of two (k = 2) and SNP densities of 30K, 15K, 7K, and 1K. (DOCX)

S2 Table Average breed composition for Canchim (C1, C2, C3, and CA), MA genetic group (MA), Charolais (CH), Nelore (NE), and Indubrasil (IB) considering k = 3 and SNP densities of 30K, 15K, 7K, and 1K. (DOCX)

S1 Fig Cross-validation errors obtained from ADMIXTURE analyses for number of populations (k) from one to five. (TIFF)

S1 File Supporting data. (RAR)
