# Supplementary material for: Study on the introgression of beef breeds in Canchim cattle using single nucleotide polymorphism markers
Source: PLoS One. 2017 Feb 9;12(2):e0171660. doi: 10.1371/journal.pone.0171660 (PMC5300224; doi:10.1371/journal.pone.0171660)
Supplement: S1 Table — (DOCX) [file pone.0171660.s001.docx]

**S1 Table. Average breed composition for Canchim (C1, C2, C3, and CA), MA genetic group (MA), Charolais (CH), Nelore (NE), and Indubrasil (IB) considering the number of populations of two (k=2) and SNP densities of 30K, 15K, 7K, and 1K**

| Breed | ADMIXTURE | |  | STRUCTURE | |  | Regression | |
| --- | --- | --- | --- | --- | --- | --- | --- | --- |
|  | 30K | | | | | | | |
|  | CL1 (min-max) | CL2 (min-max) |  | CL1 (min-max) | CL2 (min-max) |  | CL1 (min-max) | CL2 (min-max) |
| C1 | 0.26 (0.24-0.28) | 0.74 (0.72-0.76) |  | 0.26 (0.24-0.28) | 0.74 (0.72-0.76) |  | 0.35 (0.30-0.39) | 0.65 (0.61-0.70) |
| C2 | 0.25 (0.09-0.33) | 0.75 (0.67-0.91) |  | 0.25 (0.08-0.33) | 0.75 (0.67-0.92) |  | 0.36 (0.12-0.48) | 0.64 (0.52-0.88) |
| C3 | 0.26 (0.19-0.33) | 0.74 (0.67-0.81) |  | 0.25 (0.18-0.32) | 0.75 (0.68-0.82) |  | 0.35 (0.24-0.43) | 0.65 (0.57-0.76) |
| CA | 0.27 (0.17-0.32) | 0.73 (0.68-0.83) |  | 0.26 (0.16-0.32) | 0.74 (0.68-0.84) |  | 0.39 (0.24-0.51) | 0.61 (0.49-0.76) |
| MA | 0.24 (0.18-0.41) | 0.76 (0.59-0.82) |  | 0.24 (0.17-0.41) | 0.76 (0.59-0.83) |  | 0.33 (0.20-0.59) | 0.67 (0.41-0.80) |
| CH | 0.01 (0.00-0.05) | 0.99 (0.95-1.00) |  | 0.00 (0.00-0.05) | 1.00 (0.95-1.00) |  | 0.01 (0.00-0.09) | 0.99 (0.91-1.00) |
| NE | 1.00 (0.93-1.00) | 0.00 (0.00-0.07) |  | 1.00 (0.92-1.00) | 0.00 (0.00-0.08) |  | 0.99 (0.93-1.00) | 0.01 (0.00-0.07) |
| IB | 0.91 (0.82-0.96) | 0.09 (0.04-0.18) |  | 0.91 (0.82-0.96) | 0.09 (0.04-0.18) |  | 0.94 (0.88-1.00) | 0.06 (0.00-0.12) |
|  | 15K | | | | | | | |
| C1 | 0.25 (0.24-0.26) | 0.75 (0.74-0.76) |  | 0.25 (0.24-0.25) | 0.75 (0.75-0.76) |  | 0.33 (0.29-0.38) | 0.67 (0.62-0.71) |
| C2 | 0.25 (0.07-0.32) | 0.75 (0.68-0.93) |  | 0.24 (0.06-0.31) | 0.76 (0.69-0.94) |  | 0.34 (0.11-0.48) | 0.66 (0.52-0.89) |
| C3 | 0.26 (0.19-0.34) | 0.74 (0.66-0.81) |  | 0.25 (0.18-0.33) | 0.75 (0.67-0.82) |  | 0.35 (0.22-0.49) | 0.65 (0.51-0.78) |
| CA | 0.26 (0.15-0.33) | 0.74 (0.67-0.85) |  | 0.26 (0.14-0.32) | 0.74 (0.68-0.86) |  | 0.38 (0.22-0.50) | 0.62 (0.50-0.78) |
| MA | 0.25 (0.18-0.43) | 0.75 (0.57-0.82) |  | 0.25 (0.18-0.43) | 0.75 (0.57-0.82) |  | 0.33 (0.18-0.58) | 0.67 (0.42-0.82) |
| CH | 0.01 (0.00-0.05) | 0.99 (0.95-1.00) |  | 0.00 (0.00-0.04) | 1.00 (0.96-1.00) |  | 0.01 (0.00-0.10) | 0.99 (0.90-1.00) |
| NE | 0.99 (0.92-1.00) | 0.01 (0.00-0.08) |  | 1.00 (0.92-1.00) | 0.00 (0.00-0.08) |  | 0.99 (0.90-1.00) | 0.01 (0.00-0.10) |
| IB | 0.87 (0.79-0.92) | 0.13 (0.08-0.21) |  | 0.86 (0.79-0.92) | 0.14 (0.08-0.21) |  | 0.94 (0.83-1.00) | 0.06 (0.00-0.17) |
|  | 7K | | | | | | | |
| C1 | 0.21 (0.21-0.22) | 0.79 (0.78-0.79) |  | 0.20 (0.19-0.20) | 0.80 (0.80-0.81) |  | 0.28 (0.21-0.36) | 0.72 (0.64-0.79) |
| C2 | 0.20 (0.02-0.29) | 0.80 (0.71-0.98) |  | 0.19 (0.00-0.27) | 0.81 (0.73-1.00) |  | 0.30 (0.11-0.48) | 0.70 (0.52-0.89) |
| C3 | 0.21 (0.13-0.31) | 0.79 (0.69-0.87) |  | 0.19 (0.11-0.30) | 0.81 (0.70-0.89) |  | 0.33 (0.16-0.51) | 0.67 (0.49-0.84) |
| CA | 0.23 (0.11-0.32) | 0.77 (0.68-0.89) |  | 0.21 (0.08-0.31) | 0.79 (0.70-0.92) |  | 0.36 (0.16-0.57) | 0.64 (0.43-0.84) |
| MA | 0.22 (0.13-0.37) | 0.78 (0.63-0.87) |  | 0.20 (0.11-0.36) | 0.80 (0.64-0.89) |  | 0.31 (0.06-0.58) | 0.69 (0.42-0.94) |
| CH | 0.02 (0.00-0.09) | 0.98 (0.91-1.00) |  | 0.01 (0.00-0.06) | 0.99 (0.94-1.00) |  | 0.03 (0.00-0.22) | 0.97 (0.78-1.00) |
| NE | 0.99 (0.89-1.00) | 0.01 (0.00-0.11) |  | 0.99 (0.90-1.00) | 0.01 (0.00-0.10) |  | 0.98 (0.83-1.00) | 0.02 (0.00-0.17) |
| IB | 0.81 (0.75-0.86) | 0.19 (0.14-0.25) |  | 0.80 (0.74-0.86) | 0.20 (0.14-0.26) |  | 0.91 (0.66-1.00) | 0.09 (0.00-0.34) |
|  | 1K | | | | | | | |
| C1 | 0.23 (0.18-0.28) | 0.77 (0.72-0.82) |  | 0.02 (0.01-0.04) | 0.98 (0.96-0.99) |  | 0.43 (0.28-0.58) | 0.57 (0.42-0.72) |
| C2 | 0.23 (0.11-0.38) | 0.77 (0.62-0.89) |  | 0.03 (0.00-0.19) | 0.97 (0.81-1.00) |  | 0.21 (0.00-0.65) | 0.79 (0.35-1.00) |
| C3 | 0.22 (0.07-0.37) | 0.78 (0.63-0.93) |  | 0.02 (0.00-0.21) | 0.98 (0.80-1.00) |  | 0.22 (0.00-0.78) | 0.78 (0.22-1.00) |
| CA | 0.25 (0.08-0.50) | 0.75 (0.50-0.92) |  | 0.04 (0.00-0.41) | 0.96 (0.59-1.00) |  | 0.31 (0.00-0.98) | 0.69 (0.02-1.00) |
| MA | 0.27 (0.13-0.44) | 0.73 (0.56-0.87) |  | 0.05 (0.00-0.35) | 0.95 (0.66-1.00) |  | 0.29 (0.00-0.84) | 0.71 (0.16-1.00) |
| CH | 0.09 (0.00-0.30) | 0.91 (0.70-1.00) |  | 0.00 (0.00-0.08) | 1.00 (0.92-1.00) |  | 0.07 (0.00-0.49) | 0.93 (0.51-1.00) |
| NE | 0.95 (0.72-1.00) | 0.05 (0.00-0.28) |  | 0.99 (0.71-1.00) | 0.01 (0.00-0.29) |  | 0.92 (0.41-1.00) | 0.08 (0.00-0.59) |
| IB | 0.67 (0.53-0.84) | 0.33 (0.16-0.47) |  | 0.67 (0.49-0.97) | 0.33 (0.03-0.51) |  | 0.85 (0.54-1.00) | 0.15 (0.00-0.46) |

^a^CL1 (min-max) = cluster 1, assigned to *Bos primigenius indicus* (minimum and maximum values)

^b^CL2 (min-max) = cluster 2, assigned to *Bos primigenius taurus* (minimum and maximum values).
