# Supplementary material for: Study on the introgression of beef breeds in Canchim cattle using single nucleotide polymorphism markers
Source: PLoS One. 2017 Feb 9;12(2):e0171660. doi: 10.1371/journal.pone.0171660 (PMC5300224; doi:10.1371/journal.pone.0171660)
Supplement: S2 Table — (DOCX) [file pone.0171660.s002.docx]

**S2 Table. Average breed composition for Canchim (C1, C2, C3, and CA), MA genetic group (MA), Charolais (CH), Nelore (NE), and Indubrasil (IB) considering k = 3 and SNP densities of 30K, 15K, 7K, and 1K.**

| Breed | ADMIXTURE | | |  | STRUCTURE | | |  | Regression | | | |
| --- | --- | --- | --- | --- | --- | --- | --- | --- | --- | --- | --- | --- |
|  | 30K | | | | | | | | | | |  |
|  | CL1 (min-max) | CL2 (min-max) | CL3 (min-max) |  | CL1 (min-max) | CL2 (min-max) | CL3 (min-max) |  | CL1 (min-max) | CL2 (min-max) | CL3 (min-max) | |
| C1 | 0.08 (0.07-0.08) | 0.18 (0.12-0.23) | 0.75 (0.69-0.80) |  | 0.21 (0.20-0.22) | 0.18 (0.13-0.24) | 0.61 (0.56-0.65) |  | 0.35 (0.29-0.40) | 0.49 (0.45-0.53) | 0.17 (0.15-0.18) | |
| C2 | 0.07 (0.00-0.13) | 0.19 (0.00-0.53) | 0.74 (0.47-0.93) |  | 0.20 (0.03-0.28) | 0.20 (0.00-0.55) | 0.60 (0.42-0.76) |  | 0.32 (0.18-0.46) | 0.51 (0.36-0.76) | 0.17 (0.06-0.30) | |
| C3 | 0.07 (0.01-0.13) | 0.19 (0.05-0.30) | 0.74 (0.64-0.83) |  | 0.20 (0.14-0.28) | 0.19 (0.06-0.31) | 0.60 (0.52-0.67) |  | 0.33 (0.18-0.49) | 0.51 (0.39-0.61) | 0.16 (0.05-0.30) | |
| CA | 0.06 (0.01-0.12) | 0.09 (0.00-0.39) | 0.84 (0.60-0.97) |  | 0.22 (0.11-0.28) | 0.10 (0.00-0.40) | 0.68 (0.49-0.79) |  | 0.33 (0.20-0.48) | 0.48 (0.33-0.63) | 0.19 (0.06-0.30) | |
| MA | 0.07 (0.02-0.20) | 0.27 (0.00-0.42) | 0.66 (0.54-0.80) |  | 0.19 (0.12-0.34) | 0.27 (0.00-0.42) | 0.54 (0.44-0.66) |  | 0.32 (0.18-0.56) | 0.51 (0.22-0.66) | 0.16 (0.04-0.33) | |
| CH | 0.00 (0.00-0.04) | 0.98 (0.92-1.00) | 0.01 (0.00-0.08) |  | 0.00 (0.00-0.04) | 0.99 (0.93-1.00) | 0.01 (0.00-0.07) |  | 0.02 (0.00-0.12) | 0.97 (0.88-1.00) | 0.02 (0.00-0.09) | |
| NE | 1.00 (0.90-1.00) | 0.00 (0.00-0.02) | 0.00 (0.00-0.08) |  | 1.00 (0.91-1.00) | 0.00 (0.00-0.02) | 0.00 (0.00-0.07) |  | 0.98 (0.82-1.00) | 0.01 (0.00-0.08) | 0.03 (0.00-0.19) | |
| IB | 0.88 (0.76-0.94) | 0.01 (0.00-0.04) | 0.12 (0.05-0.21) |  | 0.90 (0.80-0.95) | 0.01 (0.00-0.04) | 0.10 (0.05-0.17) |  | 0.05 (0.00-0.24) | 0.00 (0.00-0.04) | 0.96 (0.76-1.00) | |
|  | 15K | | | | | | | | | | |  |
| C1 | 0.08 (0.07-0.08) | 0.22 (0.18-0.25) | 0.70 (0.66-0.74) |  | 0.16 (0.16-0.16) | 0.20 (0.16-0.24) | 0.64 (0.61-0.68) |  | 0.33 (0.30-0.36) | 0.50 (0.48-0.51) | 0.17 (0.15-0.19) | |
| C2 | 0.07 (0.00-0.14) | 0.21 (0.01-0.53) | 0.72 (0.47-0.92) |  | 0.15 (0.00-0.22) | 0.19 (0.00-0.53) | 0.66 (0.47-0.83) |  | 0.30 (0.13-0.44) | 0.56 (0.40-0.82) | 0.14 (0.00-0.27) | |
| C3 | 0.08 (0.01-0.14) | 0.19 (0.06-0.31) | 0.73 (0.64-0.80) |  | 0.16 (0.09-0.24) | 0.18 (0.02-0.30) | 0.66 (0.58-0.74) |  | 0.32 (0.14-0.48) | 0.54 (0.41-0.66) | 0.15 (0.03-0.34) | |
| CA | 0.06 (0.00-0.15) | 0.11 (0.00-0.43) | 0.83 (0.57-0.97) |  | 0.16 (0.06-0.23) | 0.09 (0.00-0.42) | 0.75 (0.52-0.86) |  | 0.30 (0.14-0.52) | 0.52 (0.35-0.71) | 0.17 (0.04-0.35) | |
| MA | 0.10 (0.03-0.25) | 0.28 (0.04-0.44) | 0.63 (0.52-0.75) |  | 0.17 (0.11-0.34) | 0.26 (0.01-0.42) | 0.57 (0.47-0.70) |  | 0.33 (0.15-0.57) | 0.54 (0.29-0.69) | 0.13 (0.02-0.32) | |
| CH | 0.01 (0.00-0.87) | 0.97 (0.00-1.00) | 0.02 (0.00-0.15) |  | 0.01 (0.00-0.89) | 0.98 (0.00-1.00) | 0.01 (0.00-0.13) |  | 0.02 (0.00-0.17) | 0.95 (0.83-1.00) | 0.02 (0.00-0.13) | |
| NE | 0.99 (0.00-1.00) | 0.00 (0.00-1.00) | 0.01 (0.00-0.12) |  | 0.99 (0.00-1.00) | 0.00 (0.00-1.00) | 0.01 (0.00-0.10) |  | 0.97 (0.81-1.00) | 0.01 (0.00-0.12) | 0.03 (0.00-0.19) | |
| IB | 0.82 (0.73-0.88) | 0.01 (0.00-0.07) | 0.17 (0.12-0.25) |  | 0.84 (0.76-0.90) | 0.01 (0.00-0.06) | 0.15 (0.10-0.23) |  | 0.06 (0.00-0.30) | 0.00 (0.00-0.06) | 0.95 (0.74-1.00) | |
|  | 7K | | | | | | | | | | |  |
| C1 | 0.06 (0.06-0.07) | 0.23 (0.21-0.26) | 0.70 (0.67-0.73) |  | 0.00 (0.00-0.00) | 0.14 (0.12-0.16) | 0.86 (0.84-0.87) |  | 0.20 (0.08-0.32) | 0.53 (0.52-0.54) | 0.27 (0.17-0.38) | |
| C2 | 0.06 (0.00-0.14) | 0.22 (0.00-0.53) | 0.72 (0.47-0.94) |  | 0.01 (0.00-0.07) | 0.12 (0.00-0.49) | 0.87 (0.51-1.00) |  | 0.25 (0.07-0.46) | 0.60 (0.31-0.78) | 0.15 (0.00-0.31) | |
| C3 | 0.06 (0.00-0.17) | 0.19 (0.04-0.33) | 0.75 (0.67-0.84) |  | 0.01 (0.00-0.10) | 0.07 (0.00-0.23) | 0.92 (0.77-1.00) |  | 0.29 (0.13-0.47) | 0.53 (0.35-0.70) | 0.18 (0.03-0.36) | |
| CA | 0.06 (0.00-0.15) | 0.12 (0.00-0.44) | 0.82 (0.56-1.00) |  | 0.00 (0.00-0.05) | 0.04 (0.00-0.38) | 0.96 (0.62-1.00) |  | 0.28 (0.02-0.58) | 0.54 (0.32-0.77) | 0.19 (0.00-0.41) | |
| MA | 0.08 (0.00-0.23) | 0.29 (0.08-0.45) | 0.62 (0.51-0.74) |  | 0.02 (0.00-0.16) | 0.20 (0.00-0.37) | 0.78 (0.63-0.96) |  | 0.29 (0.04-0.62) | 0.56 (0.28-0.79) | 0.15 (0.00-0.36) | |
| CH | 0.01 (0.00-0.81) | 0.96 (0.05-1.00) | 0.03 (0.00-0.18) |  | 0.01 (0.00-0.82) | 0.99 (0.00-1.00) | 0.01 (0.00-0.22) |  | 0.04 (0.00-0.28) | 0.93 (0.72-1.00) | 0.03 (0.00-0.22) | |
| NE | 0.98 (0.00-1.00) | 0.01 (0.00-1.00) | 0.02 (0.00-0.15) |  | 0.99 (0.00-1.00) | 0.00 (0.00-1.00) | 0.01 (0.00-0.14) |  | 0.97 (0.74-1.00) | 0.01 (0.00-0.22) | 0.03 (0.00-0.26) | |
| IB | 0.76 (0.68-0.82) | 0.06 (0.00-0.14) | 0.18 (0.08-0.28) |  | 0.76 (0.68-0.82) | 0.02 (0.00-0.15) | 0.22 (0.05-0.32) |  | 0.07 (0.00-0.37) | 0.00 (0.00-0.05) | 0.95 (0.69-1.00) | |
|  | 1K | | | | | | | | | | |  |
| C1 | 0.21 (0.20-0.22) | 0.73 (0.69-0.78) | 0.06 (0.02-0.09) |  | 0.01 (0.00-0.02) | 0.01 (0.01-0.01) | 0.98 (0.98-0.98) |  | 0.24 (0.00-0.48) | 0.37 (0.30-0.44) | 0.39 (0.22-0.56) | |
| C2 | 0.18 (0.00-0.30) | 0.73 (0.59-0.86) | 0.09 (0.00-0.22) |  | 0.01 (0.00-0.03) | 0.04 (0.00-0.45) | 0.96 (0.55-1.00) |  | 0.17 (0.00-0.76) | 0.66 (0.24-0.94) | 0.18 (0.00-0.57) | |
| C3 | 0.15 (0.03-0.24) | 0.74 (0.60-0.88) | 0.11 (0.03-0.20) |  | 0.01 (0.00-0.07) | 0.02 (0.00-0.15) | 0.97 (0.85-1.00) |  | 0.18 (0.00-0.71) | 0.62 (0.15-1.00) | 0.19 (0.00-0.71) | |
| CA | 0.19 (0.04-0.33) | 0.71 (0.47-0.87) | 0.10 (0.00-0.24) |  | 0.01 (0.00-0.14) | 0.01 (0.00-0.48) | 0.98 (0.52-1.00) |  | 0.22 (0.00-0.91) | 0.55 (0.00-0.97) | 0.24 (0.00-1.00) | |
| MA | 0.18 (0.05-0.31) | 0.70 (0.53-0.83) | 0.12 (0.02-0.25) |  | 0.05 (0.00-0.34) | 0.25 (0.00-0.97) | 0.70 (0.01-1.00) |  | 0.26 (0.00-0.80) | 0.59 (0.10-1.00) | 0.16 (0.00-0.71) | |
| CH | 0.08 (0.00-0.51) | 0.87 (0.24-1.00) | 0.06 (0.00-0.25) |  | 0.01 (0.00-0.73) | 0.98 (0.02-1.00) | 0.01 (0.00-0.32) |  | 0.11 (0.00-0.68) | 0.80 (0.32-1.00) | 0.09 (0.00-0.64) | |
| NE | 0.56 (0.00-1.00) | 0.05 (0.00-0.95) | 0.40 (0.00-1.00) |  | 0.98 (0.00-1.00) | 0.01 (0.00-0.99) | 0.01 (0.00-0.20) |  | 0.91 (0.22-1.00) | 0.04 (0.00-0.47) | 0.09 (0.00-0.76) | |
| IB | 0.43 (0.29-0.54) | 0.31 (0.15-0.45) | 0.26 (0.17-0.33) |  | 0.66 (0.50-0.97) | 0.15 (0.00-0.50) | 0.19 (0.00-0.49) |  | 0.09 (0.00-0.45) | 0.04 (0.00-0.31) | 0.92 (0.47-1.00) | |

^a^CL1 (min-max) = cluster 1, assigned to Nelore breed (minimum and maximum values),

^b^CL2 (min-max) = cluster 2, assigned to Charolais breed (minimum and maximum values),

^c^CL3 (min-max) = cluster 3, assigned to Indubrasil breed (minimum and maximum values).
