# Supplementary figures and images for: Study on the introgression of beef breeds in Canchim cattle using single nucleotide polymorphism markers
Source: PLoS One. 2017 Feb 9;12(2):e0171660. doi: 10.1371/journal.pone.0171660 (PMC5300224; doi:10.1371/journal.pone.0171660)

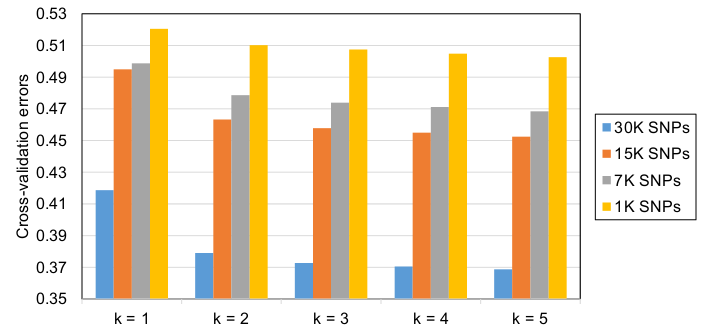

Supplement: S1 Fig — (TIFF) [file pone.0171660.s003.tiff]
